# Impact of processing techniques on donor human milk: a multi-omics and bioinformatics scoping review

**DOI:** 10.3389/fnut.2026.1768504

**Published:** 2026-03-19

**Authors:** Sergio Agudelo-Pérez, Juanita Diaz-Bruce, Camila Karduss Preciado, Sofia Yanes-Galavis, Lina Murillo Garantiva, Daniela Ortiz Peralta

**Affiliations:** Department of Pediatrics, School of Medicine, Universidad de La Sabana, Chía, Colombia

**Keywords:** bioactive components, bioinformatics, human milk banks, multi-omics analysis, human milk processing, donor human milk

## Abstract

**Introduction:**

Mothers’ milk is the optimal source of nutrition for infants. When unavailable, pasteurized donor human milk (PDHM) is the recommended alternative, particularly for premature neonates at a risk of food insecurity. However, processing methods, such as pasteurization, can alter the nutritional and bioactive composition of milk. This scoping review synthesizes current evidence from multi-omics technologies and bioinformatics to characterize the biochemical impact of processing on PDHM and identify knowledge gaps.

**Methods:**

A systematic literature search was conducted using five databases (Medline, EMBASE, Scopus, LILACS, and Web of Science) in accordance with the PRISMA Extension for Scoping Reviews (PRISMA-ScR) guidelines. Observational and descriptive studies characterizing the proteome, glycome, lipidome, or metabolome of pasteurized milk using high-resolution analytical techniques such as mass spectrometry or nuclear magnetic resonance spectroscopy were included. Of 304 articles screened, 18 met the inclusion criteria.

**Results:**

The processing effects varied markedly depending on the biomolecule and the technique. Holder pasteurization (HoP), the most widely used method, causes substantial degradation of key immune proteins (e.g., IgA and lactoferrin) and enzymes. In contrast, human milk oligosaccharides (HMOs) and trace elements remained relatively stable. Alternative methods, including high-temperature short-time (HTST) pasteurization and high-pressure pasteurization (HPP), more effectively preserve the bioactive components. Notably, HoP promotes the formation of Maillard reaction products and advanced glycation end products (AGEs), which may have detrimental biological effects.

**Conclusion:**

Multi-omics analyses highlighted a critical trade-off between microbiological safety and the preservation of bioactive integrity in PDHM, particularly with HoP. These technologies are essential for quantifying processing-induced alterations and for guiding the development of improved preservation strategies. Such approaches are pivotal for implementing precise nutritional interventions aimed at optimizing health outcomes in vulnerable neonatal populations.

## 1 Introduction

Mothers’ milk is universally recognized as the best food for healthy infants, premature neonates in the neonatal intensive care unit (NICU), and growing infants. There are clinical and social situations in which the mother’s milk is unavailable or insufficient to meet the child’s nutritional demands (1). Under these circumstances, the World Health Organization (WHO) recommends feeding with pasteurized donor human milk (PDHM) as the best alternative to mother’s milk and is superior to infant formula (2). The use of PDHM not only benefits premature or pathologically conditioned neonates, but also healthy infants at risk of food insecurity (1). It is estimated that approximately 800,000 infants will receive a PDHM in 2020 (3). The benefit of feeding premature neonates PDHM is mainly observed in the reduction of necrotizing enterocolitis (NEC) and sepsis episodes, in addition to improving oral feeding tolerance (4, 5). On the other hand, healthy infants at the community level have shown adequate growth rates when they receive PDHM as a sole source or supplement for breastfeeding (6).

The PDHM is produced by the national network of human milk banks (HMBs) (7–9). These institutions play a crucial role in achieving sustainable development goals by ensuring human milk feeding and promoting the reduction of malnutrition and neonatal and infant mortality while also representing a cost-effective strategy for neonatal care (10, 11). This impact has driven their global expansion, with efforts aimed at establishing new milk banks in the low- and middle-income regions (12). Ensuring the microbiological safety of donor human milk (DHM) involves several steps: collecting milk from multiple donors, processing and pooling to homogenize the nutrient content, pasteurization, and storage (11, 13). Furthermore, its composition is determined by factors such as gestational age, lactation stage, and the diet of the donating mothers (14). This can result in variability in the final compounds of PDHM compared to native milk, leading to a change in nutritional components (13). Because the health benefits of human milk are provided by its nutritional components and bioactive molecules, it is necessary to characterize the composition of PDHM (15).

Traditional analytical techniques are insufficient to capture this complexity. Therefore, the need to expand knowledge in this field is recognized, and new techniques based on multi-omics approaches such as proton nuclear magnetic resonance spectroscopy (H NMR) and mass spectrometry (MS) are being employed. These high-resolution technologies have the potential to provide a more detailed understanding of the specific composition of PDHM, influence of different variables, and determine its safety (16, 17). Their application in infant nutrition opens new avenues to improve health outcomes, thus offering the opportunity to enhance neonatal nutritional care (15, 18–20).

Given these considerations, this scoping review aimed to systematically characterize the nutritional composition and bioactive molecules of pasteurized donor human milk using multi-omics and bioinformatics analyses from high-throughput platforms such as H NMR and MS, and to identify existing knowledge gaps to guide future research and processing innovations.

## 2 Materials and methods

This scoping review was conducted in accordance with PRISMA Extension for Scoping Reviews (PRISMA-ScR) guidelines (21).

### 2.1 Literature search strategy

A literature search was conducted in June 2025 using the electronic databases Medline, EMBASE, Scopus, Latin American and Caribbean Health Sciences Literature (LILACS), and Web of Science. A “snowball” search was also carried out by identifying additional studies from the reference lists of publications selected for full-text review.

The search terms included the following keywords, synonyms, and subject indexing terms (MeSH and DeCS): milk banks, human milk banks, human milk banking, pasteurization of human milk, mass spectrometry, proton magnetic resonance spectroscopy, proteomics, metabolomics, lipidomics, oligosaccharides, and glycomics. The search strategy in PubMed was as follows: ((Milk Banks) OR (Human Milk Bank) OR (Donor Human Milk) OR (Human Milk Banking) OR (Pasteurization)) AND ((Mass Spectrometry) OR (Magnetic Resonance Spectroscopy)) AND ((Omics) OR (Proteomics) OR (Lipidomics) OR (Metabolomics) OR (Oligosaccharides) OR (Glycomics)).

### 2.2 Study eligibility criteria

Observational and descriptive studies were included, in English or Spanish, that analyzed donor human milk subjected to pasteurization in HMBs using high-resolution technology via mass spectrometry and/or proton nuclear magnetic resonance spectroscopy accompanied by separation techniques and characterized the composition in terms of nutrients and/or cellular composition (biomolecules). Literature reviews, conference abstracts, posters, letters to the editor, summaries, and *in vitro* studies were excluded.

### 2.3 Study screening and inclusion

The search and selection of studies were carried out by researchers (SA-P, JB, SG, CP, LG, and DP) in a blind and independent manner. The initial screening was performed by title and abstract using the Rayyan® web tool, where duplicate records were initially identified owing to the overlap between the consulted databases. The screening results were compared and discrepancies were resolved by consensus among the researchers. Articles identified as relevant for the scoping review were retrieved from the full text for in-depth reading by the researchers independently to determine their final inclusion. Discrepancies were resolved through consensus.

### 2.4 Data extraction and synthesis

Study characteristics were extracted using a standardized data charting form, including (a) bibliographic information: author, year of publication, and country of origin; (b) study title; (c) analytical scope; (d) pasteurization or sterilization method applied; (e) high-resolution analytical technology used; (f) bioinformatics database employed; (g) characterized component(s) (e.g., proteome, lipidome, metabolome, glycome); (h) population and/or sample analyzed; and (i) clinical characteristics of the donor–infant dyad. For the qualitative synthesis of information, categorization was planned according to the characterization of the omics component, pasteurization methodology, and the clinical and/or biological characteristics.

## 3 Results

**Figure 1:**
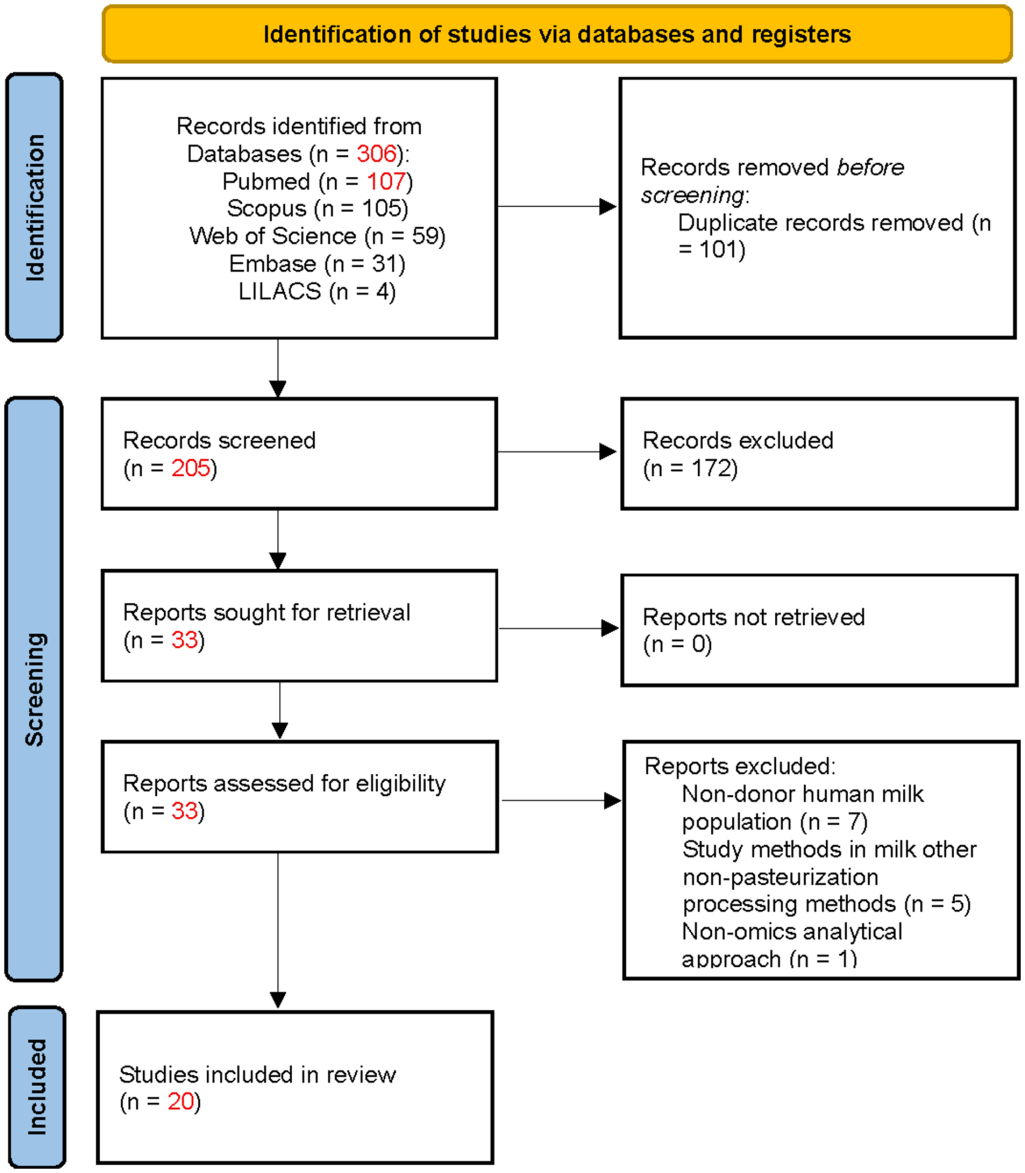
PRISMA flow diagram of the study selection process.

A total of 205 records were identified. During the initial screening, 172 documents were excluded. After full-text assessment, 13 additional studies were excluded, resulting in 20 studies included in the final analysis. The main reasons for exclusion were incorrect population, outcome, publication type, and irrelevant subject matter (Figure 1).

Supplementary Table S1 summarizes the main characteristics of the studies included in this scoping review, detailing their analytical scope, pasteurization or sterilization methods, high-resolution technologies, bioinformatics tools, main components analyzed, study population, and principal findings.

The included studies will be published between 2011 and 2025. The United States was the most frequent country of origin, followed by France, and other regions. Most investigations have employed proteomics either as a single approach or combined with other omics strategies, while metabolomics, lipidomics, and glycomics have also been represented across studies. Several studies have adopted multi-omics designs that integrate two or more analytical layers to provide a broader characterization of donor human milk composition.

High-resolution analytical platforms are predominantly based on mass spectrometry coupled with liquid or gas chromatography, frequently complemented by advanced bioinformatics processing pipelines. The primary objective of most studies has been to evaluate the compositional and functional changes in pasteurized donor human milk using different processing techniques. Additional variables evaluated across studies included the lactational stage, storage conditions, and, in some cases, simulated gastrointestinal digestion models or differences between milk pools and individual donor samples.

### 3.1 Human Milk treatment: pasteurization/ sterilization techniques

The main objective of most studies has been to evaluate the effect of processing methods on the nutritional and bioactive components of DHM, including proteins, lipids, carbohydrates, trace elements, and nucleotides. Various sterilization techniques have been investigated, which can be classified as thermal, pressure, irradiation, and freeze-drying methods. The aim was to understand the differences resulting from the implementation of each technique and compare the effects between them.

Thermal methods have been studied extensively. This includes holder pasteurization (HoP), which consists of heating the milk to 62.5 °C for 30 min; it served as the reference method and was evaluated in 18 of the 20 studies, either alone (22–26) or compared with alternative techniques (27–32). Other techniques included high-temperature short-time (HTST) pasteurization, which was evaluated in four studies, two of which compared it with HoP (33–35), flash heating (FH), low-temperature pasteurization (VAT), thermo-ultrasonication (TUS), and retort sterilization (RTR) (27–32).

Among the nonthermal methods, high-pressure processing (HPP) was the most prominent, evaluated in six studies, always in comparison with HoP techniques (27–29, 36, 37). The irradiation method refers to UV-C treatment, which has been evaluated in a small number of studies, where changes in selected human milk components have been reported (36). Freeze-drying was performed in two studies (30, 38).

Overall, these studies compared alternative processing strategies with HoP to assess differences in the microbiological safety and preservation of milk biocomponents.

### 3.2 Clinical characteristics of donors

Most studies have analyzed milk obtained from healthy donors with term pregnancies. However, some studies included specific clinical populations.

Van Keulen et al. (27) conducted a prospective case–control study comparing milk from mothers who recovered from COVID-19 with milk from healthy donors. They evaluated viral neutralization capacity and IgA levels following HoP and HPP processing. Both methods showed reductions in total IgA compared to raw milk, with a greater reduction observed after HoP. The virus neutralization capacity differed significantly between pasteurized milk (HoP and HPP) and unpasteurized milk. This functional activity is better preserved after high-pressure processing (HPP) than after Holder pasteurization (HoP). While HPP-treated milk showed a neutralization capacity comparable to that of unpasteurized milk, HoP resulted in a significant reduction in virus neutralization capacity when compared with both HPP and unpasteurized milk.

Only a limited number of studies have included preterm-related samples or explicitly reported the gestational age composition. Alves et al. (25) analyzed 60 milk samples from mothers of preterm infants and compared them with donor milk from term births. Higher concentrations of trace elements such as Ba, Cu, Mo, Se, and Zn were observed in preterm milk regardless of pasteurization status.

Meredith-Dennis et al. (31) compared the macronutrient concentrations across HoP, VAT, and RTR treatments. The HoP samples consisted of pooled milk, containing 11% preterm milk and 89% term milk. No significant differences were observed according to gestational age, whereas differences were identified according to processing technique.

### 3.3 Multi-omic characterization of pasteurized donor human Milk (PDHM)

The overall impact of processing techniques on the major bioactive components identified across omics domains is summarized in Table 1.

**Table 1:** Summary of the impact of thermal and non-thermal processing techniques on key bioactive components of human milk.

Bioactive component	HoP	HTST	HPP	Freeze-drying
Immunological proteins (IgA, Lactoferrin)	Severe reduction (20%–80%) (36, 43)	Improved preservation vs. HoP (33, 35)	High preservation (>80%) (36)	High preservation (>90%) (38)
Lysozyme	Variable, from preservation to reduction (36)	Preservation >90% (35)	High preservation (36)	High preservation (38)
BSSL	Almost total inactivation (>66%–99%) (33, 34)	Partial preservation (~60%) (33)	Partial preservation (~62%) (36)	High preservation (38)
HMOs	Total concentration stable, possible changes in specific profiles (30, 31)	Not evaluated	Concentration and profile stable (37)	Concentration and profile stable (30)
Maillard products / AGEs	Significant increase (37)	Not evaluated	No significant increase (37)	No significant increase (30)
Trace elements (Fe, Zn, Cu)	General stability, no significant changes (24, 25)	Not evaluated	General stability (28)	Not evaluated
Fatty acids (General profile)	Contradictory findings: from reduction to stability (26, 31)	General stability (34)	General stability (29)	Not evaluated

HoP, Holder pasteurization; HTST, high-temperature short-time pasteurization; HPP, high-pressure processing; IgA, immunoglobulin A; BSSL, bile salt–stimulated lipase; HMOs, human milk oligosaccharides; AGEs, advanced glycation end-products; Fe, iron; Zn, zinc; Cu, copper.

The table synthesizes the general findings of the studies included in this review. The percentages are approximate and may vary depending on the specific study conditions. The references correspond to those cited in the text.

#### 3.3.1 Proteomic analysis

Proteomic evaluation is the most frequently used analytical focus across studies. Kontopodi et al. (36) conducted two investigations to compare the pasteurization methods. In one study, HoP was associated with reduced levels of IgA, lactoferrin, and lysozyme compared to non-thermal methods such as HPP, TUS, and UV-C. In a subsequent study (35), HTST and FH were compared with HoP, showing preservation of IgA and lysozyme with HTST, whereas lactoferrin levels decreased across thermal treatments.

Nebbia et al. (33) and Baro et al. (34) compared HoP with HTST and reported similar overall protein degradation patterns, although higher retention of immunoglobulins and lactoferrin was observed in HTST. Kim et al. (39) evaluated VAT, UHT, and RTR and reported a higher preservation of total protein with VAT, with progressively lower concentrations observed with UHT and RTR. Dennis et al. (31) reported comparable findings, with RTR associated with the greatest reductions in immunoglobulins and VAT, showing higher preservation of protein concentrations.

Recent proteomic evidence has supported these findings. A dynamic *in vitro* digestion study using pooled donor milk subjected to HoP or HPP demonstrated that HPP preserved the structural integrity of key bioactive proteins and improved the release of essential amino acids during simulated gastrointestinal digestion, whereas HoP resulted in greater protein denaturation (40).

Protein expression also varies according to the lactation stage. Won-Ho et al. (38) reported no significant differences between 15 and 60 days postpartum in freeze-dried samples, whereas another study reported progressive decreases in protein expression up to 6 months of lactation (22). Variability in amino acid content between milk banks has also been reported, although pooling reduces the inter-bank variability (41).

#### 3.3.2 Glycomic analysis: stability of HMOs and the hidden risk of the Maillard reaction

Carbohydrate analyses have primarily focused on human milk oligosaccharides (HMOs), using mass spectrometry (30–32, 37). Overall, the total HMO concentration and composition were not significantly affected by HoP or freeze-drying, although they varied between mothers and throughout lactation.

However, Meredith-Dennis et al. (31) found that, although the total carbohydrate content was stable, specific HMO profiles (fucosylated, sialylated, and non-fucosylated neutral) varied significantly depending on the pasteurization technique (HoP vs. VAT vs. RTR). For example, the concentration of HMOs in the HoP samples was almost double that obtained using other techniques. This finding suggests that the thermal impact can selectively affect different HMO families.

Perhaps the most critical finding in glycomic analysis comes from a study by Marousez et al. (37) when comparing HoP and HPP, they observed that both methods preserved HMOs similarly. However, HoP caused a significant increase in the formation of three Maillard reaction products (final glycation products). This phenomenon, resulting from the heat-induced reaction between sugars and proteins, not only reduces the nutritional value of milk but also generates compounds potentially harmful to the neonate.

Salcedo et al. (32) reported no significant changes in gangliosides after HoP and UHT, whereas HTST was associated with an increased total ganglioside concentration during storage.

#### 3.3.3 Lipidomic analysis: a landscape of contradictory findings

The lipidomic findings were heterogeneous across studies. Doménech et al. conducted a detailed analysis of 36 fatty acids and observed that HoP significantly altered the lipid profiles (26). They reported a decrease in the concentration of saturated fatty acids (−25%), polyunsaturated fatty acids (PUFAs, −18%), and monounsaturated fatty acids (MUFAs, −12%). Although essential fatty acids such as arachidonic acid (ARA) and docosahexaenoic acid (DHA) were not affected, linoleic acid did show a decreased.

Marousez et al. (29), Meredith-Dennis et al. (31), and Baro et al. (34) evaluated techniques such as HoP, HTST, VAT, RTR, and HPP, and concluded that, in general, fat content, major short-chain fatty acids, and bile salt-stimulated lipase (BSSL) were adequately preserved. Although they did not focus on gangliosides, Marousez et al. (29) and Baro et al. (34) reported no significant changes in short-chain fatty acid profiles or BSSL activity with HPP and HTST, respectively, compared to the almost complete degradation of BSSL associated with HoP. This discrepancy suggests that while HoP may have a generalized negative impact, alternative techniques might preserve certain components of the lipidome or that the impact varies depending on the specific type of fatty acid analyzed.

Enzymatic activity is a key aspect of lipidome. BSSL is fundamental to fat digestion in neonates with immature pancreatic systems. Nebbia et al. confirmed that HoP destroyed 66% of BSSL, whereas HTST destroyed only 39% (33). Despite this difference, they found no differences in triglyceride hydrolysis during *in vitro* digestion, suggesting the existence of compensatory mechanisms.

#### 3.3.4 Metabolomic analysis: stability of micronutrients

Metabolomic analyses have been used to evaluate trace elements, nucleotides, lipids, and broader metabolic pathways. Overall, the findings indicate that pasteurization, whether Holder pasteurization (HoP) or high-pressure processing (HPP), has a limited impact on the concentration of most essential trace elements, such as iodine, iron, molybdenum, selenium, zinc, and copper (24, 25). Although a slight decrease in iron was observed, it is not considered clinically relevant, and the major source of variability appears to be the initial iron concentration in the donor mother’s milk (24).

Mateos-Vivas et al. (28) reported that HoP increased nucleotide monophosphate levels, likely due to the heat-induced degradation of polymeric nucleotides such as RNA, whereas HPP did not produce significant changes. Importantly, nucleotide concentrations remained stable after 6 months of frozen storage, regardless of the processing method used.

Beyond micronutrients, broader metabolomic analyses have shown that processing techniques can induce global metabolic alterations. Doménech et al. (26) identified reductions in the pathways related to steroid hormone biosynthesis and unsaturated fatty acid metabolism after HoP, suggesting a wider metabolic impact. Similarly, untargeted metabolomic studies have demonstrated that both HoP- and pressure-based techniques can modify lipid-related pathways and phospholipid profiles, although the magnitude of the change varies depending on the processing conditions (42).

## 4 Discussion

This scoping review integrates the current evidence derived from multi-omics technologies into the composition of PDHM. The 20 analyzed studies showed that the impact of processing varied considerably depending on the technique used and the biomolecular components evaluated. To facilitate interpretation, this discussion is structured around five axes: the dilemma between safety and bioactivity, functional consequences of molecular alterations, risks derived from thermal by-products, stability of beneficial compounds, and potential for precision neonatal nutrition.

### 4.1 Microbiological safety vs. bioactive integrity

The main challenge for human milk banks (HMBs) is ensuring microbiological safety without compromising the biological value of milk. Holder pasteurization (HoP) has proven to be effective for pathogen inactivation (43). However, accumulating multi-omics evidence has consistently demonstrated a significant loss of key proteins and enzymes (Table 1), compromising the immunological and functional quality of PDHM. Alternative methods, such as HTST pasteurization and high-pressure processing (HPP), appear to better preserve structurally sensitive proteins, including immunoglobulins and lactoferrin (35, 36). Freeze-drying, as a non-thermal technique, also preserves bioactive components better, although it requires a combination of methods, such as irradiation, to ensure microbiological safety (22, 38). This shift in focus reflects that the goal of HMBs is no longer just to offer safety but to preserve the biological functionality of milk (44).

The differences observed between the thermal processing techniques are likely explained by variations in the overall thermal load (temperature–time combinations) and heat transfer dynamics. High-temperature short-time (HTST) treatments, characterized by brief exposure to elevated temperatures, appear to better preserve structurally sensitive proteins, such as immunoglobulins and lactoferrin, by limiting protein unfolding and aggregation. In contrast, low-temperature longer-time approaches, such as VAT, may maintain the total protein concentration but can still modify functional properties. More intensive techniques, such as retort sterilization, which combines high temperature and pressure, are associated with greater protein denaturation and loss of specific bioactive components. These findings suggest that both processing intensity and protein structural susceptibility are key determinants of proteomic alterations in pasteurized donor human milk.

### 4.2 Functional consequences of immunological protein loss

The reduction of IgA and lactoferrin represents not only quantitative variation but also a loss of immunological function. IgA protects mucous membranes against enteric and respiratory pathogens, whereas lactoferrin has antimicrobial, anti-inflammatory, and immunomodulatory effects. Its degradation, which is widely documented in milk treated with HoP (45), could explain why PDHM does not offer the same anti-infective protection as raw milk (46, 47). Freeze-drying, by not significantly altering proteins, allows for preservation of bactericidal activity (38). Salcedo et al. demonstrated that this technique preserves gangliosides, which are associated with antimicrobial activity. Furthermore, the preservation of enzymes, such as BSSL and lipids, facilitates the production of free fatty acids with antimicrobial properties (22). Together, these mechanisms may contribute to the reduction in infections, sepsis, and necrotizing enterocolitis in hospitalized neonates.

Beyond the loss of specific immunological proteins, an additional and largely unexplored dimension relates to how milk processing affects downstream digestive functionality. While proteomic studies included in this scoping review provide valuable information on the preservation or loss of specific bioactive proteins and enzymes after milk processing, none of the available multi-omics studies have directly assessed protein digestibility or residual proteolytic activity in pasteurized donor human milk. Current evidence is largely limited to compositional or functional evaluations prior to digestion, such as immunological activity or enzymatic function (e.g., BSSL activity), without addressing how processing-induced structural changes may influence protein hydrolysis, peptide release, or gastrointestinal digestion in infants. Consequently, the impact of milk processing on protein digestibility remains an important and underexplored knowledge gap, highlighting the need for future studies that integrate proteomics with *in vitro* digestion models or functional simulations.

### 4.3 Maillard reaction products and AGEs

In addition to bioactive loss, the formation of potentially harmful compounds via thermal treatment is a critical consideration. Marousez et al. (37) demonstrated that HoP promotes the formation of Maillard reaction products, in contrast to HPP. This reaction reduces protein digestibility, destroys essential amino acids, and generates allergenic compounds. Advanced glycation end products (AGEs) derived from this reaction are associated with oxidative stress, inflammation, and tissue damage. Neonatal exposure to exogenous AGEs, particularly in premature infants with immature antioxidant systems, is a substantial theoretical concern (13).

Maillard reaction products such as furosine, furfurals, and advanced glycation end products (AGEs) are not only markers of thermal damage, but have also been associated with reduced nutritional quality and activation of oxidative and inflammatory pathways linked to chronic diseases (48). Both endogenous and dietary AGEs contribute to the total AGE pool in the body, and elevated levels have been associated with pathological conditions largely mediated by inflammatory mechanisms (49).

In addition to their biochemical formation, specific Maillard-derived compounds identified in pasteurized donor human milk, including AGEs such as carboxymethyl-lysine, have been linked to oxidative stress and immune modulation. In neonates, particularly preterm infants with immature antioxidant and detoxification systems, exposure to exogenous AGEs could theoretically contribute to intestinal inflammation, endothelial dysfunction, and altered immune responses. Although direct clinical evidence remains limited, these molecular findings underscore the need for further investigation of the biological implications of thermal processing.

### 4.4 Preservation of neuroprotective and prebiotic compounds

Some bioactive components such as HMOs are highly resistant to processing. Their preservation after HoP is relevant because they function as prebiotics, immune modulators, and neuroprotective factors, and contribute to NEC prevention (50–52). Nevertheless, structural alterations in specific HMO profiles after processing have been reported (31), which may have functional implications given the structure-specific biological activities of these molecules (51).

Gangliosides and sphingolipids, which are essential for neuronal membrane integrity, synaptogenesis, and myelination, are preserved in most processing techniques. Evidence from experimental and clinical studies suggests that adequate early life exposure to these lipids supports cognitive development and visual function (53, 54). Therefore, their preservation in processed milk may have direct translational relevance, particularly in preterm infants at risk of neurodevelopmental impairment. However, whether the quantitative or structural changes induced by processing translate into measurable clinical outcomes remains to be established.

### 4.5 Variability of PDHM and personalized nutrition

PDHM composition exhibits substantial variability driven by both processing techniques and maternal factors, including lactation stage, gestational age, diet, genetics, and ethnicity (16, 19). Pooling donor milk is commonly used to standardize the composition and dilute potential contaminants; however, this practice may also attenuate biologically meaningful individual differences (41). In this context, multi-omics technologies offer an opportunity to move toward precision nutrition by enabling the identification of specific compositional profiles and facilitating the targeted allocation of donor milk according to clinical needs. For example, batches enriched in immunological proteins could be prioritized for infants at high risk of infection, whereas distinct lipidomic profiles may better support neurodevelopment. Such an approach could reposition PDHM as a personalized therapeutic resource, rather than as a uniform nutritional substitute.

Importantly, current multi-omics evidence on pasteurized donor human milk does not allow stratification according to donor characteristics, such as maternal age or comorbidities, including metabolic conditions, such as diabetes or hypertension. Although these determinants have been widely investigated in unprocessed human milk, they remain largely unexplored in the context of processed donor milk. This gap is particularly relevant in settings in which maternal metabolic disorders are prevalent and may influence milk composition. Therefore, future multi-omics studies integrating donor clinical profiles with processing variables are needed to support more precise and context-specific nutritional strategies for vulnerable infants.

This scoping review has several limitations, including the relatively small number of available studies, methodological heterogeneity across analytical platforms, and limited representation of milk from mothers of preterm infants. Future research should prioritize standardized comparative designs, the inclusion of clinically vulnerable populations, and the development of rapid, robust, and scalable analytical approaches that can be feasibly implemented in milk bank settings.

## 5 Conclusion

The multi-omics characterization of pasteurized donor human milk indicated that processing exerts heterogeneous effects on its nutritional and bioactive components, largely depending on the technique applied and the molecular domain evaluated. The most consistent alterations were observed in the proteome, where conventional Holder pasteurization was associated with a reduction in key immunological proteins. In contrast, human milk oligosaccharides and trace elements demonstrate relative stability across processing methods.

However, emerging evidence on the formation of Maillard reaction products during thermal processing introduces important considerations regarding the biological implications of donor-milk treatment. High-resolution multi-omics approaches provide critical insights into these compositional changes and represent an essential tool for guiding the optimization of processing strategies and future development of precision nutrition approaches aimed at improving outcomes in vulnerable neonatal populations.
